# Fixing Formalin: A Method to Recover Genomic-Scale DNA Sequence Data from Formalin-Fixed Museum Specimens Using High-Throughput Sequencing

**DOI:** 10.1371/journal.pone.0141579

**Published:** 2015-10-27

**Authors:** Sarah M. Hykin, Ke Bi, Jimmy A. McGuire

**Affiliations:** 1 Department of Integrative Biology, 3101 Valley Life Sciences Building, University of California, Berkeley, California, United States of America; 2 Computational Genomics Resource Laboratory (CGRL), California Institute for Quantitative Biosciences (QB3), University of California, Berkeley, California, United States of America; 3 Museum of Vertebrate Zoology, 3101 Valley Life Sciences Building, University of California, Berkeley, California, United States of America; Natural History Museum of Denmark, University of Copenhagen, DENMARK

## Abstract

For 150 years or more, specimens were routinely collected and deposited in natural history collections without preserving fresh tissue samples for genetic analysis. In the case of most herpetological specimens (i.e. amphibians and reptiles), attempts to extract and sequence DNA from formalin-fixed, ethanol-preserved specimens—particularly for use in phylogenetic analyses—has been laborious and largely ineffective due to the highly fragmented nature of the DNA. As a result, tens of thousands of specimens in herpetological collections have not been available for sequence-based phylogenetic studies. Massively parallel High-Throughput Sequencing methods and the associated bioinformatics, however, are particularly suited to recovering meaningful genetic markers from severely degraded/fragmented DNA sequences such as DNA damaged by formalin-fixation. In this study, we compared previously published DNA extraction methods on three tissue types subsampled from formalin-fixed specimens of Anolis carolinensis, followed by sequencing. Sufficient quality DNA was recovered from liver tissue, making this technique minimally destructive to museum specimens. Sequencing was only successful for the more recently collected specimen (collected ~30 ybp). We suspect this could be due either to the conditions of preservation and/or the amount of tissue used for extraction purposes. For the successfully sequenced sample, we found a high rate of base misincorporation. After rigorous trimming, we successfully mapped 27.93% of the cleaned reads to the reference genome, were able to reconstruct the complete mitochondrial genome, and recovered an accurate phylogenetic placement for our specimen. We conclude that the amount of DNA available, which can vary depending on specimen age and preservation conditions, will determine if sequencing will be successful. The technique described here will greatly improve the value of museum collections by making many formalin-fixed specimens available for genetic analysis.

## Introduction

The primary goal of natural history museums is to preserve a biological record of the natural world for scientific study [1]. Museum collections have long provided geographic, morphological, and life history data for biologists. During approximately the past four decades, museums have also become the repositories of choice for tissue samples for molecular genetic analyses, especially for non-model organisms. These tissue collections are the necessary source materials for a tremendous diversity of biological studies. Given that the vast majority of museum specimens were collected before the advent of molecular genetics and routine collection of tissue samples, researchers have long been interested in developing protocols that would allow for the successful collection of historical DNA (hDNA) sequence data directly from museum specimens, even when properly prepared tissue samples were not available [2]. The traditional taxon-specific methods by which museum specimens have been prepared were a critical factor in this effort. Organisms prepared as study skins or dry preps, including birds, mammals and herbarium specimens are not typically exposed to formalin during preparation, and have been found to be highly amenable to hDNA data collection using traditional Sanger sequencing [3–5]. Indeed, hDNA extraction and Sanger sequencing from museum skins of birds, mammals and ancient human remains has not only been used in numerous routine studies, but also made possible molecular studies of extinct species [6–8], as well as studies of historical populations spanning both time and space [9–11].

Recent advances in High-Throughput Sequencing (HTS) data collection have revolutionized molecular genetic studies by making it possible to rapidly and efficiently obtain data sets composed of hundreds or even thousands of loci. Unsurprisingly, HTS sequencing efforts using museum study skins as source material have been very successful, and it is now possible to obtain not just DNA sequence data sets but genomic-scale DNA sequence data sets from these samples [12,13]. Of course many museum specimens are not routinely prepared as study skins or dry preps, but rather are formalin-fixed and stored in ethanol as fluid specimens. The organisms most often prepared in this manner include fish, amphibians, reptiles, and various invertebrate taxa. The extraction of usable DNA sequence data from these materials has proven much more challenging and, indeed, largely intractable. Studies that have successfully recovered DNA sequences from formalin-fixed samples generally obtained only short fragments (often for mitochondrial genes) by stitching together very small sequence fragments (typically just 50–100 base pairs in length) painstakingly obtained using custom-designed primers for each short read. Thus, developing effective and consistent protocols for the successful extraction and sequencing of historical formalin-fixed samples has been elusive, leaving millions of formalin-fixed museum specimens collected over the course of decades largely unavailable for molecular genetic analysis. Furthering progress in unlocking this potential treasure trove is the primary objective of this study.

Formalin-fixation of specimens damages DNA in three ways: (1) fragmentation, (2) base modification, and (3) cross-linkage within the DNA itself or between DNA and proteins [14–17]. Though DNA is still present, stretches that can be sequenced are heavily and randomly fragmented, posing a challenge for Sanger-sequencing techniques. Sanger sequencing relies on targeting specific regions of the genome to accurately copy long (typically 300–1500 bp) stretches of DNA, rendering the random and fragmented formalin-fixed DNA particularly unsuitable. Illumina high-throughput sequencing, however, typically sequences as few as 50–150 contiguous nucleotides per read, and can produce several hundred million such reads spanning an entire genome. This, in conjunction with the bioinformatics techniques for assembling reads and aligning them to a reference genome, makes the Illumina platform a promising one for sequencing DNA from formalin-fixed tissues.

Attempts to extract DNA from formalin-fixed museum specimens (FFMS) [16,18] have been successful, but the methods were relatively destructive to specimens—often requiring removal and destruction of skeletal elements—and labor-intensive when using a Sanger-sequencing platform. More recently, attempts to sequence formalin-fixed, paraffin-embedded (FFPE) human and cancer-cell lines have been successful using HTS [19–21]. However, there are notable differences in the protocols employed for formalin-fixing and paraffin-embedding cell cultures versus formalin-fixation of museum specimens. In particular, cell lines are usually exposed to a 2%—10% formalin solution for mere minutes (typically 20 min or less) before paraffin embedding [15,19], whereas FFMS are injected with and then soaked in 10% (or more) formalin solution for anywhere from 12 hours to several weeks. In addition, the vast majority of FFMS are prepared under conditions known only to the researchers who prepare them, as these data are not routinely recorded. In addition to the age of the specimen, this introduces a wide range of variables (e.g. light exposure, temperature, formalin concentration, whether or not the formalin was buffered) that could affect DNA quality and sequencing success for any particular specimen.

In this study, we attempted to develop an extraction and HTS protocol for DNA from formalin-fixed, ethanol-preserved herpetological museum specimens. We conducted a parallel set of comparative extraction experiments on two formalin-fixed, ethanol-preserved Anolis carolinensis specimens: one collected and preserved ~100 years ago, the other ~30 years ago. We subsampled liver, leg muscle, and tail-tip from each specimen and performed DNA extractions following two different protocols to determine (i) which tissue yielded a larger quantity of DNA, and (ii) which protocol performed better for each tissue type. This was followed by Illumina HTS of the best extraction for each specimen. After processing the resulting data for quality, the sequences were aligned to the A. carolinensis genome to determine if accurate and phylogenetically informative sequence data could be recovered. In this paper, we report the results of these experiments and outline a minimally-destructive protocol for obtaining phylogenetically informative sequence data from formalin-fixed museum specimens.

## Materials and Methods

### Tissue Collection and DNA Extraction

We subsampled liver, leg muscle, and tail-tips from two specimens of Anolis carolinensis from the University of California Museum of Vertebrate Zoology (MVZ) at Berkeley. These specimens were MVZ 214979, collected from Louisiana and prepared in 1985, and MVZ 43405, collected from Louisiana and prepared in 1917. These specimens were chosen for subsampling according to two criteria: each was formalin-fixed and preserved in ethanol, and both were large enough, approximately 80–100 mm snout-to-vent length (SVL), to allow subsampling of approximately 0.05 g of leg muscle tissue from the inguinal region without severely damaging the specimen. Subsampling was performed with standard, non-sterile, steel forceps and scissors. Tissues were stored separately in 70% ethanol in sterile 1.5 ml microcentrifuge tubes. Samples ranged in mass from 0.01 g to 0.5 g.

To limit potential contamination, extractions were performed in a room used exclusively for DNA extraction from historical specimens. There had been no previous extractions of Anolis performed in this laboratory space. The two extraction protocols performed were adapted from [19,22,23]. Both protocols begin with a series of ethanol washes followed by treatment in a heated alkali buffer solution. While heat and alkali degrade DNA, limited exposure to a combination of both has been demonstrated to be effective in breaking protein-DNA cross-linkages caused by formalin-exposure [16]. The hot alkali treatment was either followed by a phenol-chloroform extraction [16], or extraction using a standard Qiagen kit [22]. For extraction of tail-tips, we used a protocol for decalcification of formalin-fixed skeletal elements [23], followed by phenol-chloroform extraction. The set of extraction protocols that we tested are provided in the Supporting Information S1 File. After extraction, we quantified DNA yield for each extraction using a Nanodrop 1000 (Thermo Fisher Scientific Products) to measure the concentration of nucleic acids, a Qubit 2.0 fluorometer (Invitrogen, Life Technologies) to measure the concentration of double-stranded DNA, and an Agilent Bioanalyzer 2100 low-sensitivity chip (Aligent Technologies, Inc.) with DNA standards at 15 and 1500 bp to quantify DNA concentration and fragment size.

### Library Preparation and Sequencing

The extraction that yielded the largest quantity of high-quality DNA for MVZ 214979 was from liver tissue using the phenol-chloroform protocol (provided as S2 File). This was prepared for sequencing using the standard TruSeq protocol for DNA (Illumina, Paired-End Sample Preparation Guide, document # 1005063 Rev. D) and NEB (New England Biolabs # E6006s) reagents. We also prepared a library for MVZ 43405 from the phenol-chloroform extraction of liver despite its failure to yield measurable amounts of quality (i.e. double-stranded) DNA. We modified the Illumina protocol for both extractions following [24] to account for the fragmented nature of formalin-fixed DNA by omitting the initial DNA fragmentation step. Instead, we proceeded immediately to end-repair, adenylation of the 3’ end of DNA fragments, and adapter ligation according to the Illumina protocol. We used Agencourt Ampure XP (Beckman Coulter) magnetic-bead purification for nucleotide recovery and purification between steps in the Illumina protocol. This was followed by 16 PCR amplification cycles using Phusion PCR High Fidelity master mix (NEB, F-531S). After bead purification of the PCR’d libraries, they were analyzed for quantity and quality of DNA present on an Agilent Bioanalyzer 2100 low-sensitivity chip with two replicates for each library. The library for the older specimen, MVZ 43405, did not contain a sufficient amount of library product and underwent an additional six cycles of PCR amplification, followed by purification and re-analysis. We then performed 100-bp paired-end Illumina sequencing, pooling both samples on one lane of a HiSeq2000 at the Vincent J. Coates Genomics Sequencing Laboratory (Q3B, University of California, Berkeley)

### Data processing

Upon receipt of raw data, pre-processing and alignment largely followed [24] as outlined below, with the following exceptions: Bowtie2 [25] was used instead of Bowtie [26] for contaminant filtration; Bowtie2 and Novoalign (www.novocraft.com) were used for alignment to the Anolis carolinensis genome (Anocar2.0, downloaded from the UCSC Genome Browser, http://watson.compbio.iupui.edu/cgi-bin/hgGateway); and updated versions of in-house scripts (https://github.com/MVZSEQ/) were employed throughout the process.

**Fig 1:**
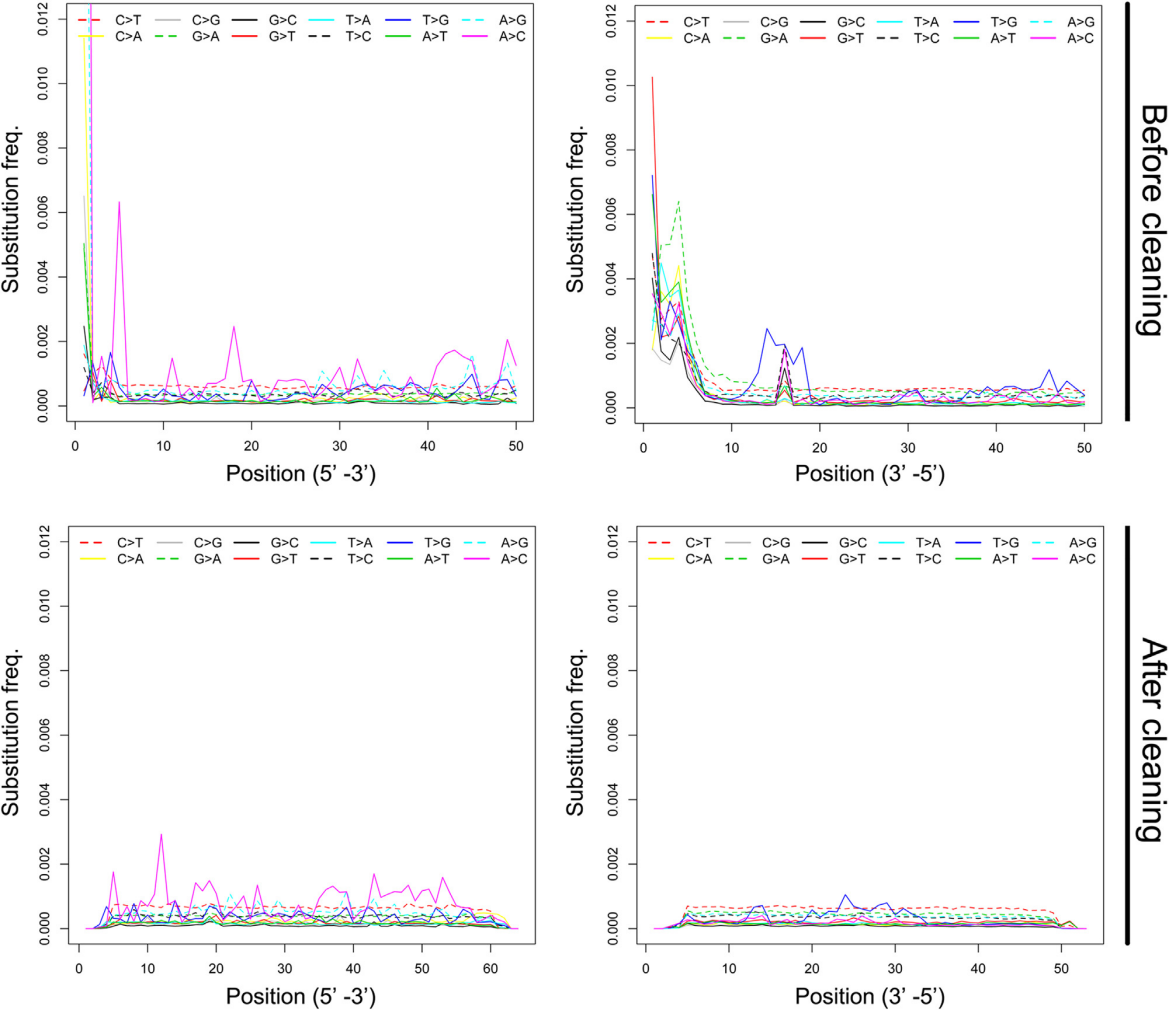
Patterns of mismatches in MVZ 214979 sequences. The frequencies of the 12 types of mismatches (y-axis) are plotted as a function of distance from the 5′ and 3′ ends of the sequence reads (x-axis). The frequencies of each mismatch type are coded in different colors and line patterns. ‘After cleaning’ shows mismatch frequencies after deleting the first 50 bp form the end of each read.

DNA recovered from ancient and museum historic specimens is often characterized by various types of postmortem nucleotide damage (e.g. [8,27,28]), and formalin-fixation can cause base modifications in the DNA fragment [15]. The specimens used in this study had been fixed in formalin for at least 30 years, thus damage to the DNA could be the result of post-mortem denaturation or subsequent exposure to formalin. To inspect potential base misincorporation in sequence reads, we first aligned the untrimmed raw paired-end reads against the Anolis carolinensis reference genome with Bowtie2. By parsing the SAM output, we generated base mismatch frequency plots by plotting the frequency of all 12 possible mismatches against distance from 5′ and 3′ ends of reads, respectively. We observed a sharp increase in mismatch frequencies of almost all types at both ends, and particularly at the 3’ end of reads (Fig 1). We then performed multiple rounds of trimming from both 5′ and 3′ ends of reads, until the frequencies of all 12 types of mismatches were relatively constant and similar along post-trimmed reads (Fig 1). As a final trimming step, we removed 36 bp from the forward reads (6 bp from 5′ and 30 bp from 3′ end) and 47 bp from the reverse reads (17 bp from 5′ end and 30 bp from 3′ end). To evaluate the quality of sequence reads before and after cleaning and trimming, SAMtools [29] and an in-house script were used to estimate empirical error rates, measured as the percentage of mismatched bases out of the total number of aligned bases in the mitochondrial genome [12].

The hard-trimmed raw sequence data were then re-processed to remove exact duplicate reads, adaptors, and low-quality sequences, and to merge overlapping paired-end reads following [24] and [30] using in-house scripts. To remove reads that might result from contamination by organisms other than Anolis, we aligned all adaptor-trimmed reads to the human (hg19) and Escherichia coli (NCBI st. 536) genomes using Bowtie2 [25]. We assumed that reads aligning to these genomes represented contamination and removed them from our data. After cleanup, we mapped the resulting paired-end reads to the *Anolis* reference genome using Novoalign, then applied SAMtools to check mapping efficiency and depth. All cleaned data, including paired-end and unpaired reads, were de novo assembled using ABySS [31] and individual assemblies were generated under a wide range of k-mers as in [32]. We used cd-hit-est [33], Blat [34], and CAP3 [35] to merge raw assemblies and reduce redundancy in our libraries. Contiguous sequences (contigs) less than 200 bp were removed. The resulting contigs were mapped to the *Anolis* reference genome using the BLASTn program [36]. To evaluate coverage of the mitochondrial genome, we mapped cleaned reads to the mitochondrial reference genome of Anolis carolinensis and used SAMtools to reconstruct the coding sequence of the mitochondrial genome from the MVZ 214979 library.

To evaluate if we had accurately recovered phylogenetically useful sequence data, we extracted the consensus sequence from reads mapping to the mitochondrial genome. We aligned our inferred complete mitochondrial sequence to the Anocar2.0 reference genome to assess sequence similarity. We then aligned the NADH dehydrogenase subunit 2 (ND2) sequence recovered from our formalin fixed sample to that available for the *Anolis* genome, as well as to ND2 data from NCBI for an outgroup, Oplurus cyclurus, and eight additional *Anolis* species, including the putative sister taxon of *A*. *carolinensis*, *A*. *porcatus*, and three other close relatives, *A*. *brunneus*, *A*. *allisoni*, and *A*. *smaragdinus* (NCBI IDs: OCU39585, AB218960, AY263042, KJ954109, AF337807, AY902412, AY296151, AY902417, and AY296195). We based this analysis on ND2 alone because this gene is widely available for *Anolis* species. We then estimated the phylogeny for this alignment using maximum likelihood under the GTR+I+G model in PAUP (version 4.0a142) [37] and calculated bootstrap values using maximum likelihood with 100 replicates, also under the GTR+I+G model in Garli [38].

## Results

### DNA extraction and library preparation

We were able to extract DNA from both specimens of Anolis carolinensis, however only phenol-chloroform extraction of liver tissue from MVZ 214979 yielded enough high-quality DNA for Illumina sequencing. According to Nanodrop analysis, concentrations of nucleic acids were generally higher in liver extractions (S1 Table), and according to Qubit quantification, extractions by either phenol-chloroform or Qiagen kit from muscle and tail-tips yielded insufficient quantities of double-stranded DNA for either specimen to proceed with library preparation. According to Qubit quantification, the phenol-chloroform extraction of liver for MVZ 214979 had a DNA concentration of 26.4 ng/μl, but the Qiagen and tail-tip extractions of this specimen and all extractions of MVZ 43405 failed to yield measurable amounts of double-stranded DNA. The Bioanalyzer results were consistent with Qubit quantifications: the phenol-chloroform extraction of liver from MVZ 214979 had a DNA concentration of 27.81 ng/μl, and the Qiagen extraction of MVZ 214979 had a DNA concentration of 1.51 ng/μl. The phenol-chloroform extraction of MVZ 43405 showed a concentration of 0.27 ng/μl, and all other extractions failed to show detectable amounts of DNA. We elected to proceed with library preparation of the phenol-chloroform liver extraction of both specimens to see if we could obtain usable data from MVZ 43405 despite the poor quantification values. Bioanalyzer results for the MVZ 214979 library showed peaks at ~120 bp and ~240 bp (S1 Fig), and a library concentration of 5.69 ng/μl and 7.62 ng/μl (average of two replicates = 6.66 ng/μl). Bioanalyzer results for MVZ 43405’s library showed unusual oscillations between ~150 bp and ~410 bp, and a final library concentration of 0.68 ng/μl and 3.04 ng/μl (average of two replicates = 1.86 ng/μl).

### Sequencing and data pre-processing

Sequencing results showed 9.51 billion base pairs (Gbp) for MVZ 214979 and 16.29 Gbp for MVZ 43405. After data cleanup, alignment to the reference genome for MVZ 43405 indicated extremely high PCR duplication (97.5%) and thus low diversity and a low unique mapping rate (0.23%). The mismatch frequency plot for MVZ 43405 indicated that, of the reads mapped to the reference genome, all types of mismatches across the length of reads showed extremely uneven distributions, making hard-trimming impossible (S2 Fig). For this reason, we concluded that sequencing of MVZ 43405 had failed, and these data were excluded from further analyses.

For the more recently collected sample, MVZ 214979, pre-processing resulted in removal of 64% of reads as duplicates. This is a larger fraction of the data set than is typical, even for hDNA [12], but we attribute this to the relatively low amount of starting DNA that we then PCR-amplified. Contamination by E. coli or Homo sapiens represented 0.27% of reads, and after filtering low quality reads, trimming adapter sequences, and merging overlapping paired-end reads, the library contained 1.27 Gbp of sequence data, accounting for 13.37% of the original data.

### Alignment to the *Anolis carolinensis* reference genome and phylogenetic informativeness

**Fig 2:**
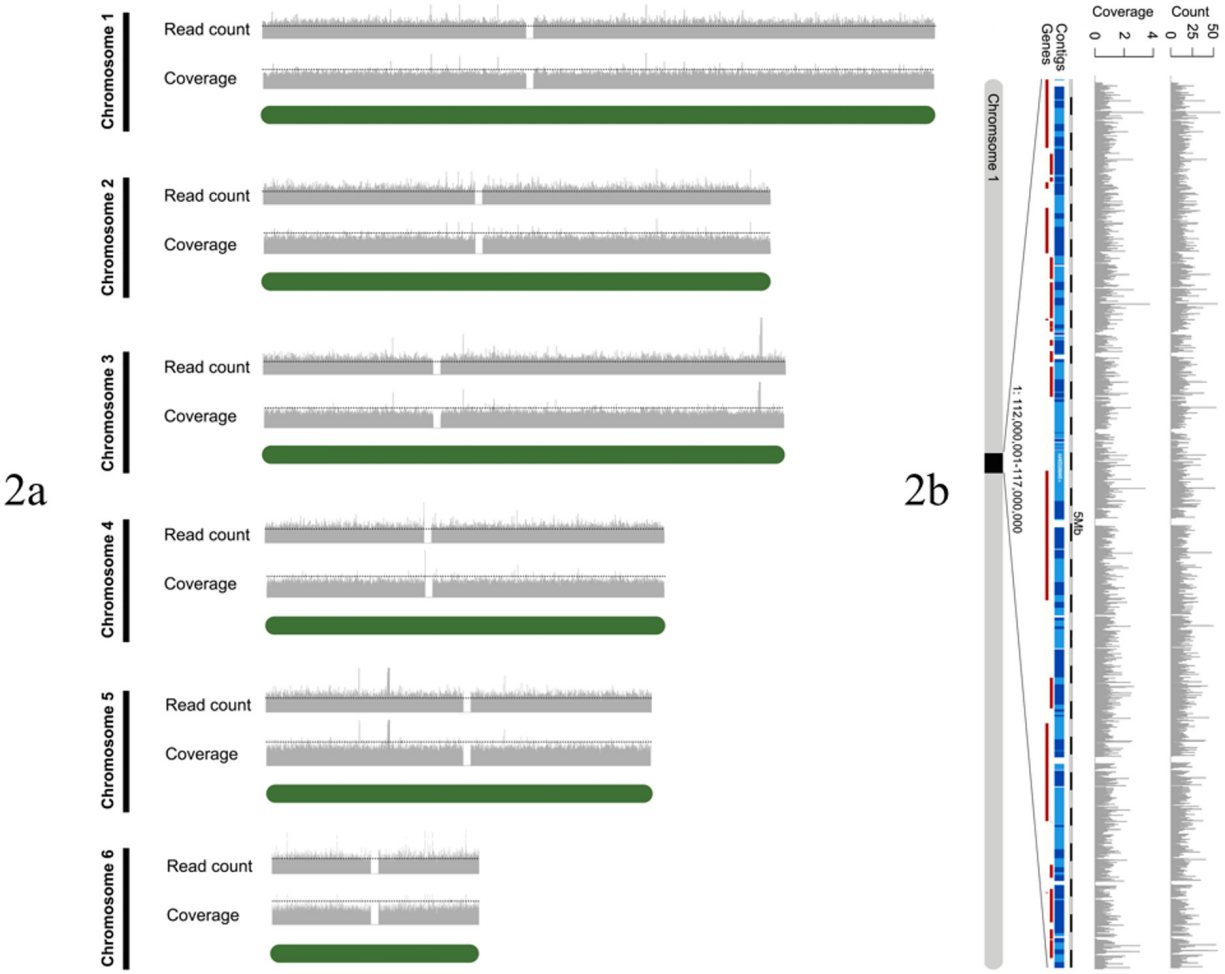
Nuclear coverage. (a) Read count and depth in 10 Kbp bins along the length of the six largest *A*. *carolinensis* chromosomes (green bars) using the MVZ 214979 library. The green line indicates a read count of 100, and coverage of 1X. (b) Read count and depth is shown in 1 Kb bins along a randomly selected 5 Mbp segment of chromosome 1 using the MVZ 214979 library.

Based on the patterns of skewed base misincorporation observed from the mismatch frequency plot, we trimmed 36 bp and 47 bp from the forward and reverse reads, respectively. After data filtration, we aligned paired-end reads and unpaired reads to the Anolis carolinensis genome using Novoalign, which resulted in the unique mapping of 70% of all cleaned reads and 72% of cleaned paired-end reads. Over the entire reference genome, 27.93% (502.7 Mb) mapped to at least one read, with and average mapping depth of 0.5X. The total amount of data aligned to the reference genome was 891.1 Mb, accounting for 9.3% of the total obtained from a half-lane’s worth of sequencing effort. Of cleaned reads, 2.9% were aligned to protein coding regions of the genome, at an average depth of 1.2X. We did not observe a bias for sequence coverage towards certain chromosomes (Fig 2a), but mapped reads were unevenly distributed within chromosomes (Fig 2b).

Raw sequence reads had an error rate of 0.61%. After hard trimming and quality filtering, the error rate decreased to 0.45%, and these final, cleaned, datasets were used for mapping, assembly, and reconstructing the complete mitochondrial genome.

Due to the highly degraded nature of DNA from MVZ 214979 and shallow sequencing depth, de novo assembly only yielded 21,394 contigs that were longer than 200 bp with an N50 of 398 bp. A total of 9342 contigs (43.67%) were aligned to the Anolis carolinensis genome with an average sequence similarity of 98.34%. Attempted *de novo* assembly of the mitochondrial genome resulted in 30 contigs representing ~75% of the mitochondrial genome (~12KB). These contigs ranged in length from 38 bp to 5157 bp. We estimated GC content in the mapped, assembled contigs to be 39.03%, comparable to the published GC content of the A. carolinensis genome of 40.30% [32].

**Fig 3:**
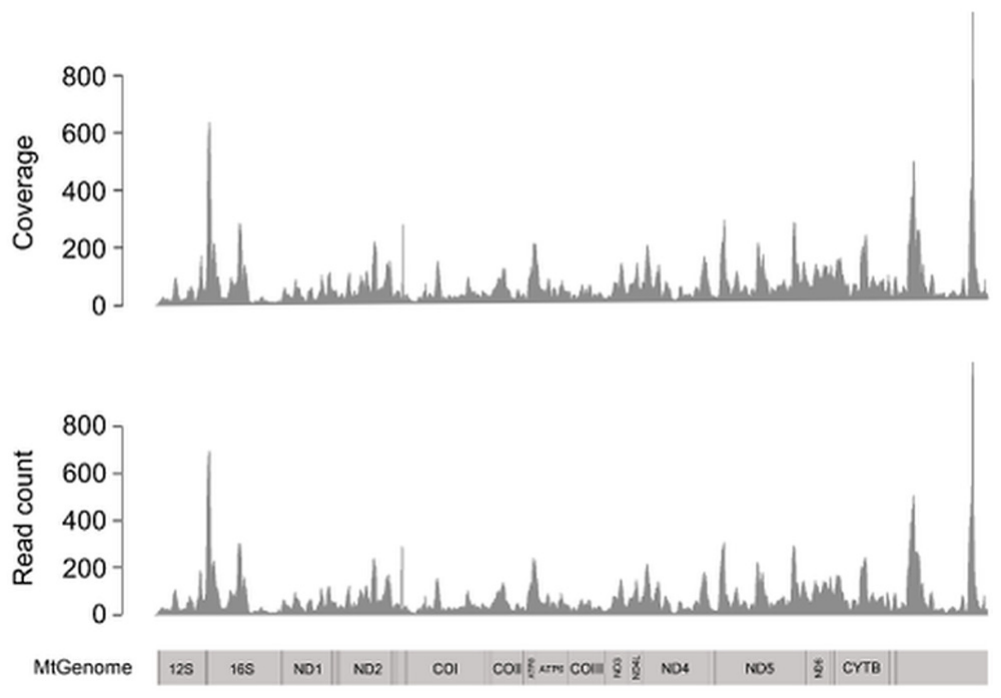
Coverage of the mitochondrial genome. Distribution of read counts (in 10 bp bins) and depth of the mitochondrial genome from the MVZ 214979. A vertebrate mitochondrial map is used for reference on the bottom to label regions of protein coding and rRNA genes. The control region is at the end of the map and is not labelled.

Our sequencing depth was too low to allow for the generation of a nuclear gene data set usable for reliable phylogenetic analysis. However, the much greater sequence depth for the mitochondrial genome was more than sufficient to recover the complete mitochondrial genome sequence, with an average depth of 57.9X. This is not surprising given the much higher per cell copy number of the mitochondrial genome as compared to the nuclear genome. As in the nuclear genome, mapped reads were unevenly distributed along the entire mitochondrial genome (Fig 3).

**Fig 4:**
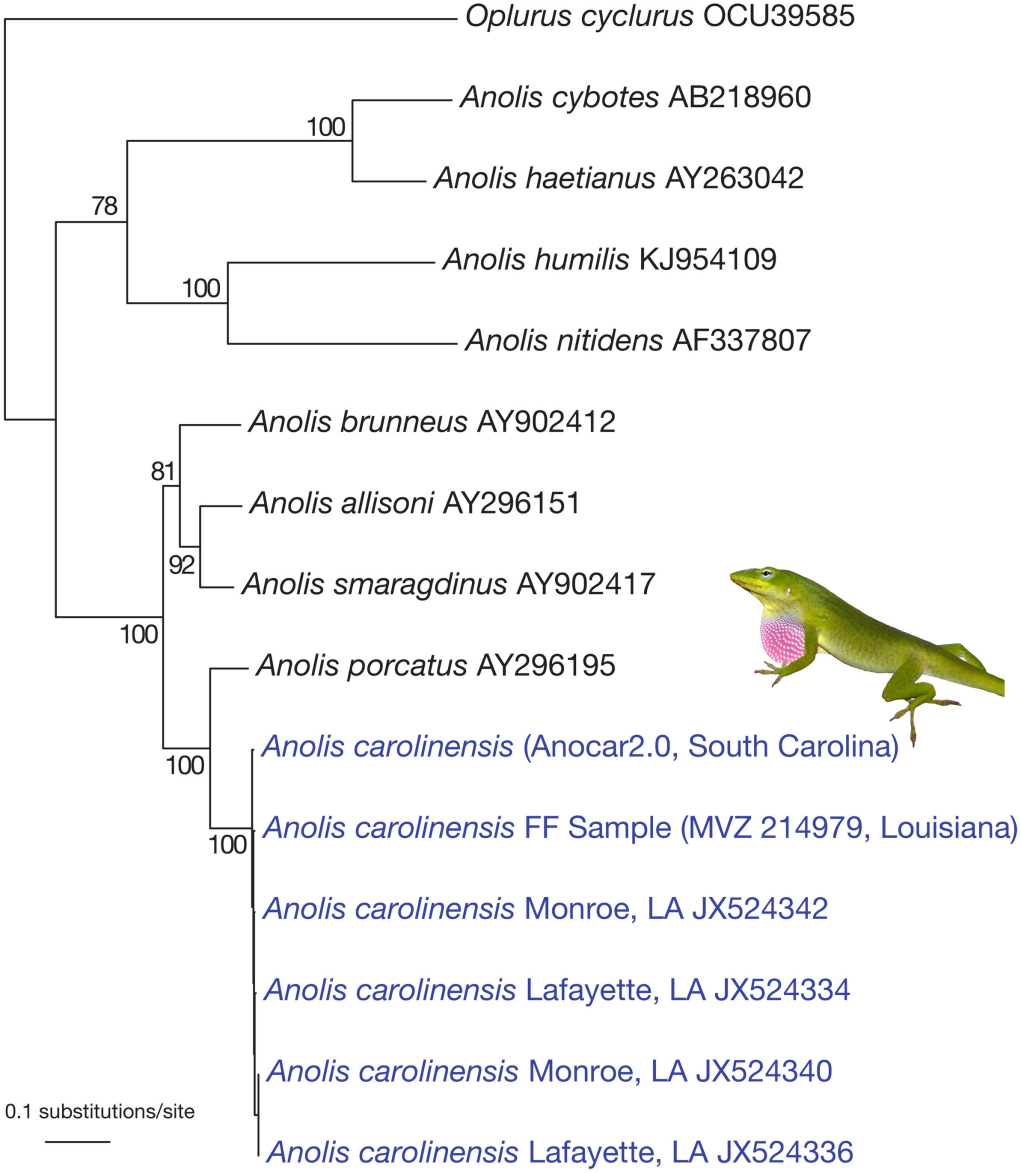
Phylogenetic inference using ND2 sequence data. Maximum-likelihood tree inferred from ND2 sequence alignment of the formalin-fixed sample (MVZ 214979), the Anocar2.0 reference genome (Anocar2.0), four *Anolis carolinensis* collected from Louisiana, USA, eight other *Anolis* species, and *Oplurus cyclurus*. *A*. *carolinensis* image printed under a CC BY license with permission of the original photographer and copyright owner J. Losos.

The total number of high quality SNPs detected between the Anolis carolinensis reference genome and this formalin-fixed specimen was 73 (0.53% sequence dissimilarity), with an average depth of 51.4X. Alignment of the 1038 bp ND2 gene from our A. carolinensis mitochondrial genome with orthologous gene regions from the reference *Anolis* genome, and to other taxa, including eight other Anolis species and an outgroup (*Oplurus cyclurus*), resulted in six SNPs between our sample and the A. carolinensis reference genome and strong support for our specimen being more closely related to A. carolinensis than to any other Anolis (Fig 4). This included A. porcatus, the sister species of A. carolinensis. In addition, the recovered ND2 sequence for MVZ 214979 was no more divergent from the reference sequence than any other *A*. *carolinensis* sequence downloaded from GenBank. This is consistent with our having recovered ND2 sequence data for MVZ 214979 with sufficient accuracy to be phylogenetically informative at the species level.

## Discussion

This study highlights both the opportunities and the challenges of obtaining genomic data from formalin-fixed museum specimens. For example, we show that phylogenetically informative DNA sequences can be generated from such specimens. Despite PCR duplications and DNA damage, our conservative approach resulted in genomic sequence data with high alignment quality for a 30-year old *Anolis carolinensis* museum specimen (MVZ 214979). In contrast, our sequencing effort failed with a 100-year old *Anolis carolinensis* specimen (MVZ 43405), which we elaborate on in greater detail below. For MVZ 214979, we obtained ~0.5X sequencing depth across the nuclear genome using half of one lane on an Illumina Hi-Seq sequencing platform. The low-coverage nuclear data were insufficient for SNP or genotype calling or to reconstruct sequence markers, especially given high error rates, but this would be remedied by greater sequencing effort (i.e., using additional sequencing lanes). Furthermore, in conjunction with our extraction protocols (S1 and S2 Files), Illumina sequencing was able to accurately recover the entire mitochondrial genome of MVZ 214979 with 57X average coverage even with the relatively modest sequencing effort employed here.

Formalin-fixation prior to DNA extraction results in extensive DNA damage as well as lower DNA yields, and sequences derived from formalin-fixed samples are likely to require special processing to account for both of these issues. For example, our raw reads required hard trimming to remove extensive base misincorporation at the ends of reads. In addition, a large percentage of our reads were discarded due to high levels of PCR duplication. The high level of PCR duplication was likely due to the low quantity of DNA available for library preparation, which then resulted in a low diversity of DNA fragments for sequencing. To obtain enough library material for sequencing, we followed the Illumina protocol’s recommendation to increase the number of PCR cycles, which likely resulted in deep sequencing of the relatively small number of unique fragments that were present in the library compared to what would be expected with a fresh tissue sample. To avoid this problem using standard library-preparation protocols, we suggest using a larger amount of starting material, and performing multiple extractions of multiple tissues with the aim of reducing the number of PCR cycles necessary during library preparation, thereby limiting the level of redundancy in final libraries. Despite requiring extensive processing as described above, the sequencing results for MVZ 214979 were comparable to those of other ancient and museum historic DNA studies for proportion of reads mapped, error rate, and percent contamination (S2 Table). The highly variable depth of coverage for both the nuclear and mitochondrial genomes (Figs 2 and 3, respectively) seen in our data is similar to that obtained in other aDNA/hDNA studies that employed non-targeted HTS [24,32]. For this reason, we suspect that this observed unevenness in coverage is more an artefact of PCR and/or sequencing and not other factors such as the composition of the *Anolis* genome or the exposure of DNA to formalin.

Since this experiment was performed, several new library preparation procedures have been developed that may be better suited to damaged or single-stranded DNA than those implemented here, and these protocols may increase the viability of otherwise marginal samples such as MVZ 43405. For example, the protocols of [39] and [40] omit the blunt-end repair step employed in this experiment, thereby maintaining the integrity of the DNA sequence at fragment-ends. Employing one of these alternate methods of library preparation and using a lower-fidelity polymerase (in order to avoid PCR bias [40,41]) might similarly improve the quality of libraries made from formalin-damaged DNA. Also, the pattern of DNA damage we observed in our formalin-fixed specimens differs in important ways from the patterns documented in prior ancient and historic DNA studies involving non-formalin-fixed specimens, suggesting that improved methods for modelling formalin damage to DNA could significantly improve sequencing success. With non-formalin-fixed samples, elevated rates of C to T misincorporated substitutions occur at the 5’ ends of DNS strands, whereas G to T transitions are elevated at the 3’ ends [28,32]. In contrast, we observed a sharp increase in mismatch frequencies of almost all types at both ends, and particularly at the 3’ ends of reads. New approximate Bayesian methods for modelling ancient and historic DNA damage, such as implemented in the program mapDamage (available at http://ginolhac.github.io/mapDamage/ [42]), will likely result in less data-loss, especially if targeted-sequencing can be used to attain sufficient coverage of regions of interest. These potential refinements of our protocol offer the potential to reduce the amount of data loss resulting from hard trimming and should be considered in future studies.

As part of this study, we compared two basic extraction protocols on three tissue types to determine what, if any, combination of protocols and tissue types would yield sufficient quantities of double-stranded DNA for successful HTS. In choosing to test liver, muscle, and tail-tip, our expectation was that tail-tip would yield the most double-stranded DNA because previous studies of fluid preserved museum specimens [18,23] found that bone tissue is likely to protect DNA from degenerative forces, and DNA can be found in larger quantities in skeletal elements. We also tested muscle tissue because excising muscle is less destructive to the specimen than taking a tail-tip, digits, or teeth. Of all tissue types considered here, liver was the only one that yielded a sufficient quantity of double-stranded DNA. This result is encouraging because, in addition to being an easy, abundant source of tissue for subsampling, taking liver is minimally destructive to fluid-preserved specimens.

MVZ 43405, collected in 1917, did not yield a sufficient quantity of high-quality DNA to warrant continuing with library preparation and sequencing under typical circumstances. Nevertheless, we attempted (unsuccessfully) to obtain genomic sequence data from this sample. Why sequencing of MVZ 43405 failed is not entirely clear. One possibility is that our attempt to systematically test alternative extraction protocols for the two specimens was the decisive factor. As noted above, only liver samples returned measureable concentrations of double-stranded DNA. For each specimen, we partitioned the liver into two subsamples for extraction, one using the Qiagen protocol and the other using the more effective modified phenol-chloroform (PC) extraction protocol. For MVZ 214979 (which was sequenced successfully), the larger piece of liver (0.46g) was extracted using PC, whereas a much smaller liver sample (0.04g) was extracted using the Qiagen protocol. This relationship was reversed for MVZ 43405, with the larger liver sample (0.33g) extracted using the less effective Qiagen protocol and a much smaller liver sample (0.017g) extracted using phenol-chloroform. Notably, the PC extraction of the much smaller liver sample taken from MVZ 43405 yielded a larger quantity of double-stranded DNA than did the Qiagen extraction of a sample ~20 times larger (S1 Table). Had we known at the outset that PC extraction would be more effective and applied that extraction protocol to the larger liver sample from the older specimen, our sequencing effort may have succeeded. Another potential explanation for the sequencing failure is the condition of the formalin used to initially fix the specimens. It is known that MVZ herpetological specimens from the early 1900s were fixed in unbuffered formalin (David B. Wake, pers. comm.). This changed around 1970, when buffering formalin became standard practice to better preserve tissues for histological studies. Because we do not know whether the two MVZ specimens were fixed with buffered or unbuffered formalin, we cannot rigorously evaluate the importance of this variable here. We can, however, note that the data obtained in our generally unsuccessful attempt to sequence MVZ 43405 are consistent with expectations for DNA damage resulting from exposure to unbuffered formalin. The sequence data that we did obtained showed an extremely uneven distribution of mismatch frequencies that could be the result of high rates of base misincorporation across the length of all reads. Paireder et al. (2013) [43] systematically compared DNA yield from tissues fixed in either buffered or unbuffered formalin for known periods of time and found that DNA yield from tissues fixed in unbuffered formalin were significantly lower than those fixed in buffered formalin after two years, which they attributed to accelerated DNA degeneration due to the higher amount of formalin per unit of fixative and to the lower pH of unbuffered formalin [43]. While the small sample size of our study prevents us from testing the effect of buffered vs. unbuffered formalin, as well as many other potential variables (e.g. temperature, sunlight exposure, conditions of long-term storage), on hDNA from museum specimens, these are important considerations and worthy of further investigation. Future studies should seek to establish heuristics on endogenous DNA content of formalin-fixed liver according to the age and preservation conditions of the specimen.

For population genetic applications, which require accurate SNP/genotype calling, two approaches may be feasible using short sequence reads derived from formalin-fixed samples. The first, a whole genome shotgun approach as employed in this study, could be sufficient to attain good alignment with the presence of a pre-existing reference genome (S2 Table), although successful application for nuclear genomic data would require greater sequencing effort per specimen than was achieved here. Lacking genomic resources, de novo genome assemblies from modern specimens of closely related species could be used for alignment given sufficient sequencing depth. Unfortunately, in cases where formalin-fixed specimens are the only available genetic material for a project, generating a sound de novo assembly seems unlikely without a prohibitive amount of sequencing effort. Not only does the sequencing depth necessary for accurate base-calling present a challenge, formalin-fixation ultimately results in severely fragmented DNA. Increasing data yield might not sufficiently improve assembly quality due to the lack of long-insert genomic libraries available for genome scaffolding. For population genetic studies in which reduced representation approaches are used, the obstacles to accurate base-calling presented by formalin-induced base misincorporation may be circumvented by targeted capture of specific genomic regions. If genomic resources are available for marker development, we suspect that exon-capture [12] or similar methods will be able to achieve the depth of coverage necessary for these applications.

Although we were able to reconstruct the complete mitochondrial genome by mapping back to the reference genome, the results of this pilot study demonstrate the difficulty in generating high quality assemblies of the nuclear genome. This was due to the inherently fragmented nature of the DNA, and the low diversity of raw sequence data. Acknowledging that Whole Genome Sequencing is not necessary for phylogenetic studies, our results suggest that target enrichment approaches such as exon-capture—successful in other museum hDNA studies of non-fluid preserved specimens [12]—could be effective for targeting nuclear genomic regions for formalin-fixed museum specimens. Attempting target enrichment was deemed outside the scope of this exploratory study, but clearly would be an excellent avenue for future work. In this vein, exon-capture requires a genomic reference with which to design probes for targeted regions, and studies lacking modern genomic resources will have to contend with the difficulty posed by de novo assembly of formalin-fixed hDNA, as addressed above. In these situations, de novo sequencing of transcriptomes and whole genomes of modern samples of the same or closely related species may be an effective alternative. The nature of formalin-induced DNA damage we observed, however, recommends against the use of restriction enzyme-based reduced representation library approaches such as RADSeq (e.g [44,45]). The likelihood of severe degradation and base modification at recognition sites in formalin-fixed DNA may cause extensive data-loss across samples.

While this method will make many formalin-fixed specimens available for genetic study, we emphasize that there are two major differences between this extraction and sequencing protocol and those used to generate data from modern samples. The first is that the amount of starting material necessary to extract sufficient quantities of double-stranded DNA for library preparation is likely to be much greater on average for formalin-fixed samples. The ratio of starting material to DNA yield, however, is not likely to be consistent between samples due to variables such as specimen age, concentration and type of formalin used for preparation, and duration of formalin exposure. Correspondingly, this method will be most appropriate for specimens for which no fresh tissue is available, such as older type specimens or those representing species that are extirpated in the wild or otherwise unavailable for resampling. We encourage researchers not only to obtain fresh tissue samples when preparing specimens (flash-frozen or preserved in an appropriate medium, such as RNALater or 95% ethanol), but also to record the protocol used for preservation and use buffered formalin when fixing specimens. The second major difference is that an informed trimming approach is necessary when working with data generated from formalin-fixed specimens. Standard HTS quality control as used for modern (fresh tissue) samples is not likely to be sufficient for formalin-fixed material.

While hurdles remain regarding the wide-scale application of HTS data collection to formalin-fixed samples, this proof-of-concept study indicates that such samples can retain extractable and usable genomic sequence data, and that these data can be mined using available short-read sequencing platforms such as Illumina. Indeed, even without applying a reduced-representation or targeted sequencing approach, we have shown that direct sequencing of low-quality formalin-fixed specimens can be used to generate substantial nuclear sequence data and a high-coverage complete mitochondrial genome. Given the large number of species that are only represented by formalin-fixed museum specimens without corresponding tissues—including species known from one or a few specimens, or known to be extinct—just the ability to generate complete mitochondrial data alone is transformative. Key questions remain to be answered, of course, including (1) which characteristics determine the quality of the historical specimens for HTS data collection (possibilities include age, whether the specimen was fixed in unbuffered formalin, how long the specimen was exposed to formalin prior to transition to ethanol, etc.), and (2) whether targeted sequencing approaches will return high quality genome-scale data for formalin-fixed specimens as they can for specimens prepared as study skins. Despite the work that still needs to be done to answer these questions and streamline genomic sequencing of formalin-fixed museum specimens, the progress made here constitutes a significant step forward. High-throughput sequencing has the potential to unlock a treasure trove of genetic and genomic information for millions of museum specimens, and bring a large fraction of many museum collections into the age of genomics.

## Supporting Information

S1 Fig Quantification of MVZ 214979. Bioanalyzer trace of MVZ 214979 library prepared from liver extraction by phenol-chloroform.
(TIFF)

S2 Fig Patterns of mismatches in MVZ 43405 sequences. The frequencies of the 12 types of mismatches (y-axis) are plotted as a function of distance from the 5′ and 3′ ends of the sequence reads (x-axis). The frequency of each mismatch type is coded in different colors and line patterns. Before cleaning the first 50 bp are shown from each end of the read.
(TIF)

S1 File Protocol for experimental extraction of hDNA from formalin-fixed herpetological museum specimens. (PDF)

S2 File Protocol for extraction of hDNA from formalin-fixed museum specimens: liver extraction by phenol-chloroform. (PDF)

S1 Table Summary of DNA yield by tissue type and extraction protocol. Extractions used in library preparation and sequencing in bold. The abbreviation “TL” indicates that amounts of DNA were too low to be quantified. DNA quantification for all assays are given in units of ng/μl.
(DOCX)

S2 Table Summary of results from ancient and historic samples sequenced on the Illumina platform. Results of this study in bold.
(TIF)
