# Supplementary figures and images for: Fixing Formalin: A Method to Recover Genomic-Scale DNA Sequence Data from Formalin-Fixed Museum Specimens Using High-Throughput Sequencing
Source: PLoS One. 2015 Oct 27;10(10):e0141579. doi: 10.1371/journal.pone.0141579 (PMC4623518; doi:10.1371/journal.pone.0141579)

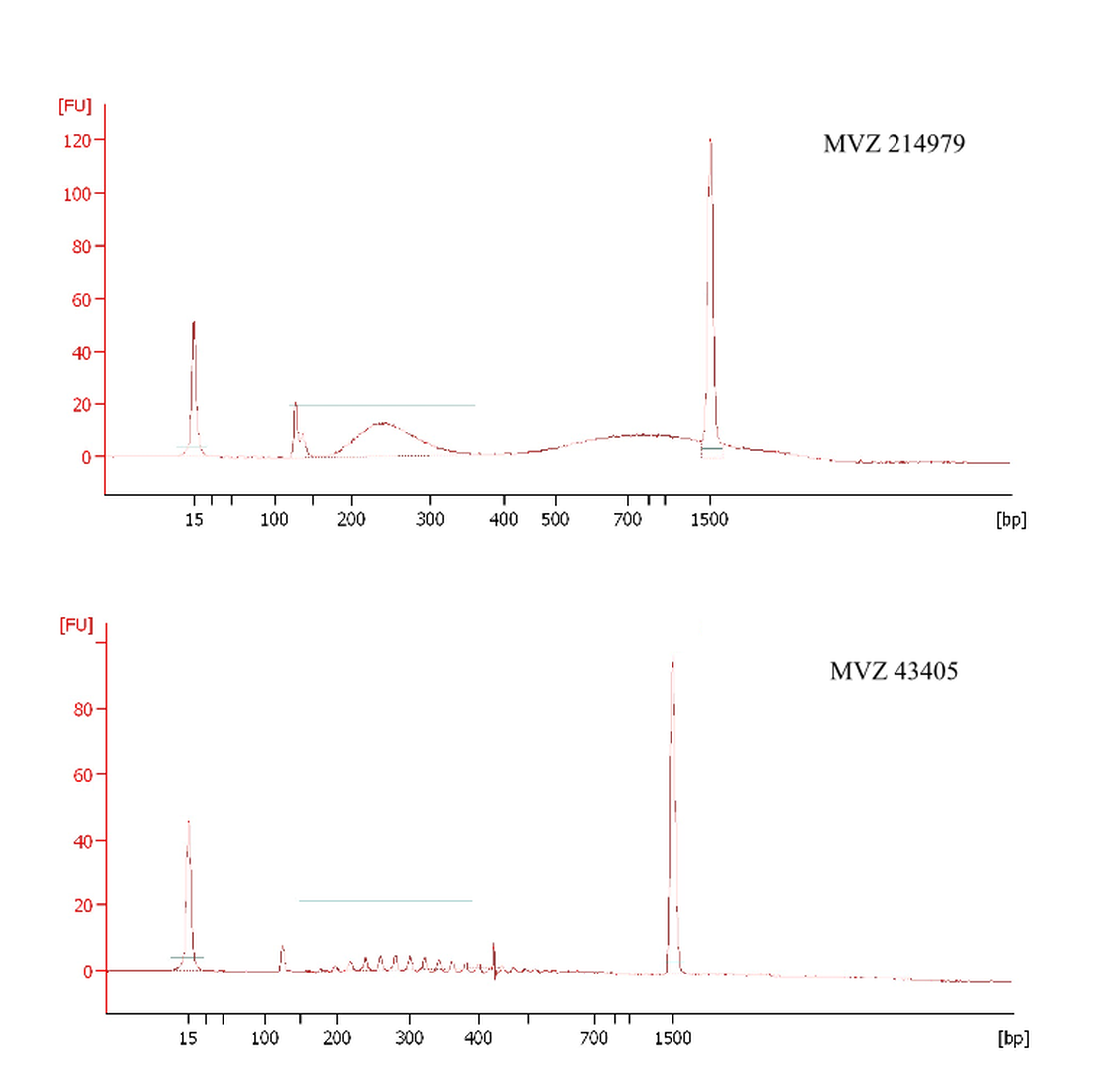

Supplement: S1 Fig — Bioanalyzer trace of MVZ 214979 library prepared from liver extraction by phenol-chloroform. (TIFF) [file pone.0141579.s001.tiff]

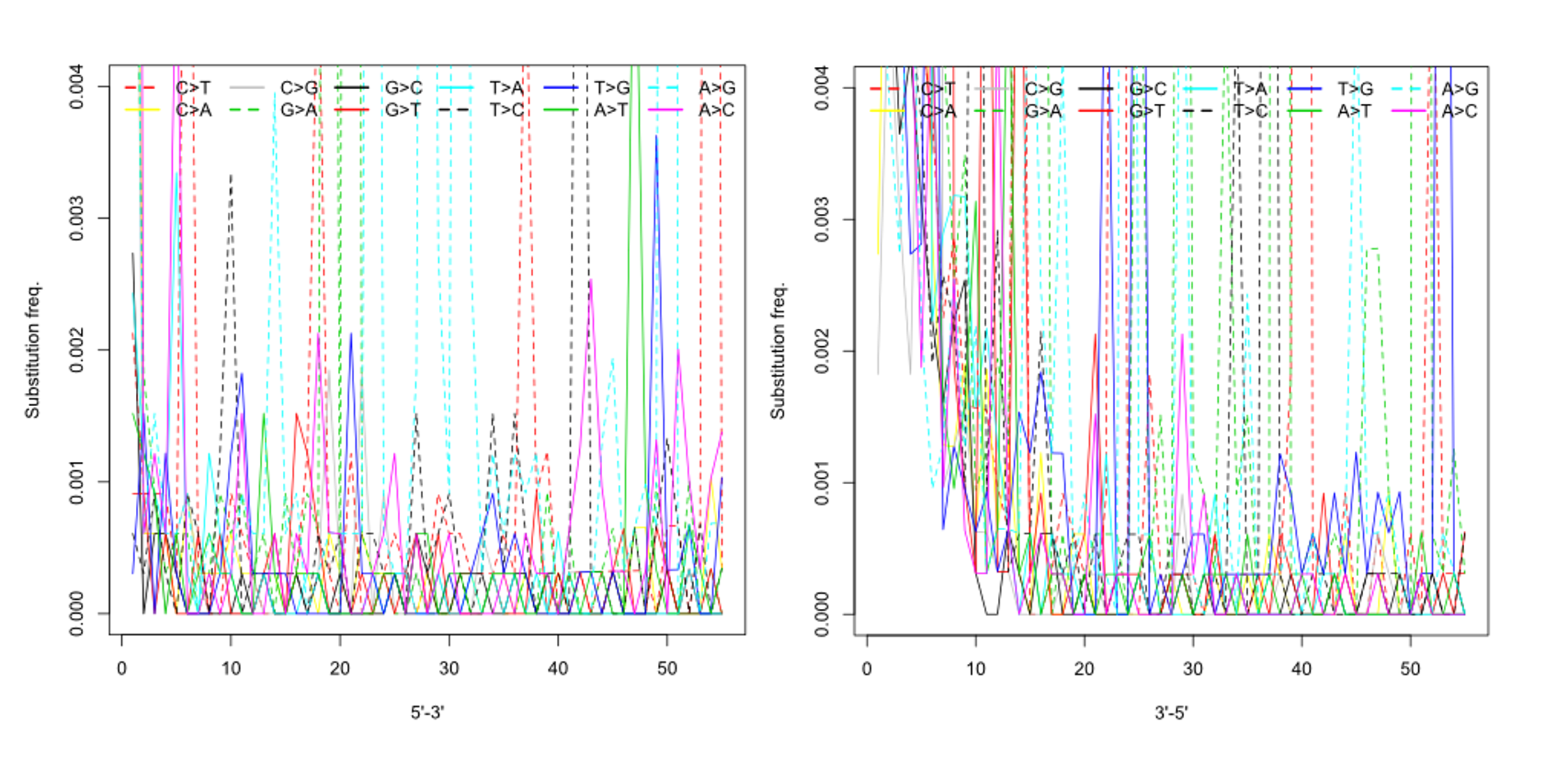

Supplement: S2 Fig — The frequencies of the 12 types of mismatches (y-axis) are plotted as a function of distance from the 5′ and 3′ ends of the sequence reads (x-axis). The frequency of each mismatch type is coded in different colors and line patterns. Before cleaning the first 50 bp are shown from each end of the read. (TIF) [file pone.0141579.s002.tif]

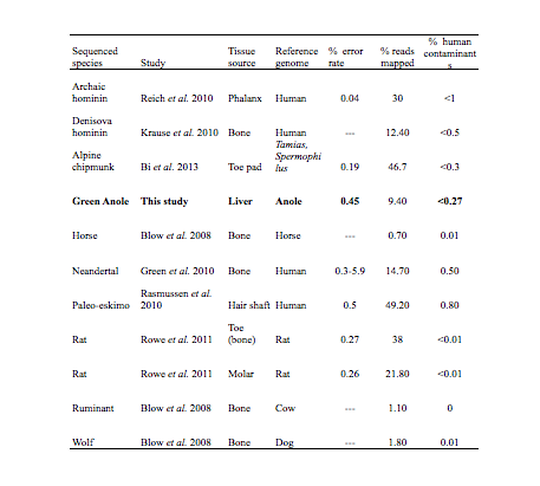

Supplement: S2 Table — Results of this study in bold. (TIF) [file pone.0141579.s006.tif]
