# Supplementary material for: Fixing Formalin: A Method to Recover Genomic-Scale DNA Sequence Data from Formalin-Fixed Museum Specimens Using High-Throughput Sequencing
Source: PLoS One. 2015 Oct 27;10(10):e0141579. doi: 10.1371/journal.pone.0141579 (PMC4623518; doi:10.1371/journal.pone.0141579)
Supplement: S1 File — (PDF) [file pone.0141579.s003.pdf]

Protocol for Experimental Extraction of hDNA from Formalin-fixed Herpetological Mueseum Specimens

SM Hykin, Adapted from Campos and Gilbert (2012), Kearney and Stuart (2004), Steinau et al. (2011)

Equipment and Reagents

Washing:
- table-top vortex
- GTE buffer (100 mM glycine, 10 mM Tris-HCL, pH 8.0, 1 mM EDTA)
- 100% ethanol
- 70 % ethanol
- sterile water

Decalcification of bone:
- mortar and pestle
- centrifuge
- agitator at room temperature
- 56 C incubator w/ rotisserie
- liquid nitrogen
- 0.5 M EDTA (pH 8.0)
- TNES buffer (10 mM Trizma Base, 100 mM NaCl, 10 mM EDTA, 2% sodium lauryl sulphate (SDS, 39 mM) DTT)
- Proteinase-K

DNA extraction

Treatment 1 – Hot alkali treatment and Qiagen DNeasy extraction (Steinau et al., 2011):
- Qiagen DNeasy Kit
- autoclave at 120 C
- incubator at 65 C
- Proteinase-K

Treatment 2 – Hot alkali and phenol-chloroform extraction (Campos and Gilbert, 2012):
- vortex, centrifuge, autoclave at 120 C, room-temp agitator
- 2 ml screw-cap O-ring tubes (important!)
- Alkali digestion buffer (0.1 M NaOH with 1% SDS solution, ~ ph 12.0, store at room temperature)
- 25:24:1 phenol:chloroform:isoamyl alcohol
- chloroform

- isopropanol
- 3 M sodium acetate, ~ pH 5.0
- (optional) DNA precipitation "carrier," e.g. Glycoblue (Ambion, Inc., Austin TX) to visualize pellet
- 85% ethanol
- TE elution buffer (10 mM Tris-HCL, 1 mM EDTA, pH 8.0)

Protocol

All steps should be conducted in a UV-hood, or in a DNA-free space. If not bone, tissue should be thinly sliced with a scalpel before proceeding.

Washing tissue: to bind excess formalin (Kearney and Stuart, 2004):
Note: Vortex tissue GENTLY after placing it in each new wash
1. wash tissue three times in 1.5 ml GTE buffer at intervals of 2 hr, 2 hr, and 12 hr.
2. wash for 1 min in 100% ethanol
3. wash for 5 min in 70% ethanol
4. wash for 10 min in sterile water

Decalcification of bone (Kearney and Stuart, 2004). If tissue is NOT bone, skip to DNA extraction.

1. Crush sample with mortar and pestle in liquid nitrogen.
2. Place sample in 1.6 ml of 0.5 M EDTA and incubate at room temperature with agitation for 48 hr.
3. Centrifuge at 8000 r.p.m. for 1 min to pellet bone fragments. Remove and discard EDTA with a pipette.
4. Perform two washes of pelleted bone fragments: add 300 µl of sterile water, centrifuge at 8000 r.p.m. for 1 minute, then remove and discard water with a pipette. Repeat.
5. Add 300 µl of TNES buffer to the washed pellet. Add 300 µl Pro-K.
6. Incubate at 56 C w/ rotisserie for 3 days, adding 300 µl of Pro-K at 24 hr intervals.

After this, the pellet will be ready for either a Qiagen kit or phenol-chloroform extraction.

DNA extraction (pick one Treatment)

Treatment 1 – Hot alkali treatment and Qiagen DNeasy extraction (Steinau et al., 2011):
1. Add 180 µl ATL lysis buffer from the DNeasy kit and incubate at 120 C for 20 min.
2. After incubation, finger-flick to ensure mixing, and centrifuge briefly to incorporate condensate.
3. Add 20 µl Pro-K and incubate at 65 C on rotisserie for 16 hr.

4. Proceed with extraction according to the standard Qiagen DNeasy protocol.

Treatment 2 – Hot alkali treatment and phenol-chloroform extraction (Campos and Gilbert, 2012)

1. Place tissue in 0.5 ml of alkali digestion buffer in a 2 ml screw-cap, O-ring tube (don't use regular microcentrifuge tubes, autoclave pressure will cause the caps to come off). DNA will start to degrade as soon as the tissue is in alkali, so do not delay the following steps.
2. Autoclave the tissue at 120 C for 25 min, do not include warm-up time. A heat-block or hot-water bath at 100 C for 40 min are alternatives, but not as effective.
3. Allow tissues to cool to room temperature. The tissue will not have fully dissolved.
4. Add 500 **µ**l 25:24:1 phenol:chloroform:isoamyl alcohol to the mixture.
5. Agitate gently at room temperature for 5 min.
6. Centrifuge for 5 min at > 10,000 x g to separate the layers.
7. Carefully remove the upper aqueous layer and add to a new tube containing 500 **µ**l chloroform. Be careful not to remove the protein-containing interface. Discard the lower phenol layer.
8. Repeat steps 5 - 6.
9. Remove the upper aqueous layer and place in a new 1.5 ml microcentrifuge tube. Discard the lower chloroform layer.
10. Add 0.6 -1 volume isopropanol and 0.1 volume 3 M sodium-acetate (~ pH 5.0). A small amount of commercial carrier solution can be added to facilitate pellet visualization following manufacturer's guidelines. Mix well.
11. Centrifuge at high speed (> 10,000 x g) for 30 min at room temperature.
12. Immediately following centrifugation, carefully decant the liquid from the tube. The DNA will have precipitated into a pellet at the bottom of the tube and may not be visible.
13. To rinse the pellet, gently add 500 - 1,000 **µ**l 85% ethanol, gently invert once, and centrifuge at 5 min at high speed.
14. Gently decant ethanol. Repeat if desired.
15. All ethanol must be removed from the pellet as any residual ethanol will inhibit downstream applications. This can be easily achieved with a small bore pipette, followed by brief incubation at a relatively high temperature (e.g., 55-75 C).
16. Re-suspend the pellet in a suitable volume of TE buffer or ddH2O (e.g., 50 - 100 **µ**l) at room temperature 10 min - overnight.

References

Campos PF and TMP Gilbert. 2012. DNA Extraction from Formalin-Fixed Material. Methods in Molecular Biology, 840:81-85.

Kearney M and BL Stuart. 2004. Repeated evolution of limblessness and digging heads

in worm lizards revealed by DNA from old bones. Proceedings of the Royal Society London Biology, 271:1677-1683.

Steinau M, Patel SS, and ER Unger. 2011. Efficient DNA extraction for HPV genotyping in formalin-fixed, paraffin-embedded tissues. The Journal of Molecular Diagnostics, 13(4): 377-381.
