# Supplementary material for: Fixing Formalin: A Method to Recover Genomic-Scale DNA Sequence Data from Formalin-Fixed Museum Specimens Using High-Throughput Sequencing
Source: PLoS One. 2015 Oct 27;10(10):e0141579. doi: 10.1371/journal.pone.0141579 (PMC4623518; doi:10.1371/journal.pone.0141579)
Supplement: S2 File — (PDF) [file pone.0141579.s004.pdf]

Protocol for Extraction of hDNA from Formalin-fixed Museum Specimens: Liver Extraction by Phenol-chloroform

SM Hykin, Adapted from Campos and Gilbert (2012), Kearney and Stuart (2004)

Equipment and Reagents

Washing:
- table-top vortex
- GTE buffer (100 mM glycine, 10 mM Tris-HCL, pH 8.0, 1 mM EDTA)
- 100% ethanol
- 70 % ethanol
- sterile water

DNA extraction – Hot alkali treatment and phenol-chloroform extraction (Campos and Gilbert, 2012):
- vortex, centrifuge, autoclave at 120 C, room-temp agitator
- 2 ml screw-cap O-ring tubes (important!)
- Alkali digestion buffer (0.1 M NaOH with 1% SDS solution, ~ ph 12.0, store at room temperature)
- 25:24:1 phenol:chloroform:isoamyl alcohol
- chloroform
- isopropanol
- 3 M sodium acetate, ~ pH 5.0
- (optional) DNA precipitation "carrier," e.g. Glycoblue (Ambion, Inc., Austin TX) to visualize pellet
- 85% ethanol
- TE elution buffer (10 mM Tris-HCL, 1 mM EDTA, pH 8.0)

Protocol

All steps should be conducted in a UV-hood, or in a DNA-free space. Tissue should be thinly sliced with a scalpel before proceeding.

Washing tissue: to bind excess formalin (Kearney and Stuart, 2004):
Note: Vortex tissue GENTLY after placing it in each new wash
1. wash tissue three times in 1.5 ml GTE buffer at intervals of 2 hr, 2 hr, and 12 hr.
2. wash for 1 min in 100% ethanol
3. wash for 5 min in 70% ethanol
4. wash for 10 min in sterile water

DNA extraction – Hot alkali treatment and phenol-chloroform extraction (Campos and Gilbert, 2012)

1. Place tissue in 0.5 ml of alkali digestion buffer in a 2 ml screw-cap, O-ring tube (don't use regular microcentrifuge tubes, autoclave pressure will cause the caps to come off). DNA will start to degrade as soon as the tissue is in alkali, do not delay the following steps.
2. Autoclave the tissue at 120 C for 25 min, do not include warm-up time. A heat-block or hot-water bath at 100 C for 40 min are alternatives, but not as effective.
3. Allow tissues to cool to room temperature. The tissue will not have fully dissolved.
4. Add 500 **µ**l 25:24:1 phenol:chloroform:isoamyl alcohol to the mixture.
5. Agitate gently at room temperature for 5 min.
6. Centrifuge for 5 min at > 10,000 x g to separate the layers.
7. Carefully remove the upper aqueous layer and add to a new tube containing 500 **µ**l chloroform. Be careful not to remove the protein-containing interface. Discard the lower phenol layer.
8. Repeat steps 5 - 6.
9. Remove the upper aqueous layer and place in a new 1.5 ml microcentrifuge tube. Discard the lower chloroform layer.
10. Add 0.6 -1 volume isopropanol and 0.1 volume 3 M sodium-acetate (~ pH 5.0). A small amount of commercial carrier solution can be added to facilitate pellet visualization following manufacturer's guidelines. Mix well.
11. Centrifuge at high speed (> 10,000 x g) for 30 min at room temperature.
12. Immediately following centrifugation, carefully decant the liquid from the tube. The DNA will have precipitated into a pellet at the bottom of the tube and may not be visible.
13. To rinse the pellet, gently add 500 - 1,000 **µ**l 85% ethanol, gently invert once, and centrifuge at 5 min at high speed.
14. Gently decant ethanol. Repeat if desired.
15. All ethanol must be removed from the pellet, as any residual ethanol will inhibit downstream applications. This can be easily achieved with a small-bore pipette, followed by brief incubation at a relatively high temperature (e.g., 55-75 C).
16. Re-suspend the pellet in a suitable volume of TE buffer or ddH2O (e.g., 50 - 100 **µ**l) at room temperature 10 min - overnight.

References

Campos PF and TMP Gilbert. 2012. DNA Extraction from Formalin-Fixed Material. Methods in Molecular Biology, 840:81-85.

Kearney M and BL Stuart. 2004. Repeated evolution of limblessness and digging heads in worm lizards revealed by DNA from old bones. Proceedings of the Royal Society London Biology, 271:1677-1683.
