# Supplementary material for: Fixing Formalin: A Method to Recover Genomic-Scale DNA Sequence Data from Formalin-Fixed Museum Specimens Using High-Throughput Sequencing
Source: PLoS One. 2015 Oct 27;10(10):e0141579. doi: 10.1371/journal.pone.0141579 (PMC4623518; doi:10.1371/journal.pone.0141579)
Supplement: S1 Table — Extractions used in library preparation and sequencing in bold. The abbreviation “TL” indicates that amounts of DNA were too low to be quantified. DNA quantification for all assays are given in units of ng/μl. (DOCX) [file pone.0141579.s005.docx]

|  |  |  |  |  |  |  |  |
| --- | --- | --- | --- | --- | --- | --- | --- |
|  | Specimen | Tissue/ Protocol | Sample weight (g) | Nanodrop | Qubit | Bioanalyzer |  |
|  | MVZ 214979 | Muscle/ Qiagen | 0.024 | 7.1 | TL | NA |  |
|  | --- | Muscle/ PC | 0.01 | 5.2 | TL | NA |  |
|  | --- | Liver/ Qiagen | 0.04 | 156.5 | 0.057 | 1.51 |  |
|  | **---** | **Liver/ PC** | **0.46** | **29.6** | **2.64** | **27.81** |  |
|  | --- | Tail-tip/ PC | 0.05 | <2.0 | NA | NA |  |
|  | MVZ 43405 | Muscle/ Qiagen | 0.01 | 4.7 | TL | NA |  |
|  | --- | Muscle/ PC | 0.01 | 10.6 | TL | NA |  |
|  | --- | Liver/ Qiagen | 0.33 | 36.7 | TL | TL |  |
|  | **---** | **Liver/ PC** | 0.017 | **5.8** | **TL** | **0.27** |  |
|  | --- | Tail-tip/ PC | 0.048 | <2.0 | NA | NA |  |
|  |  |  |  |  |  |  |  |
